# Combining Epidemiologic and Biostatistical Tools to Enhance Variable Selection in HIV Cohort Analyses

**DOI:** 10.1371/journal.pone.0087352

**Published:** 2014-01-29

**Authors:** Christopher Rentsch, Ionut Bebu, Jodie L. Guest, David Rimland, Brian K. Agan, Vincent Marconi

**Affiliations:** 1 Atlanta Veterans Affairs Medical Center, Decatur, Georgia, United States of America; 2 Emory University School of Medicine, Atlanta, Georgia, United States of America; 3 Rollins School of Public Health, Emory University, Atlanta, Georgia, United States of America; 4 Infectious Disease Clinical Research Program, Uniformed Services University of the Health Sciences, Bethesda, Maryland, United States of America; University of Athens, Medical School, Greece

## Abstract

**Background:**

Variable selection is an important step in building a multivariate regression model for which several methods and statistical packages are available. A comprehensive approach for variable selection in complex multivariate regression analyses within HIV cohorts is explored by utilizing both epidemiological and biostatistical procedures.

**Methods:**

Three different methods for variable selection were illustrated in a study comparing survival time between subjects in the Department of Defense’s National History Study and the Atlanta Veterans Affairs Medical Center’s HIV Atlanta VA Cohort Study. The first two methods were stepwise selection procedures, based either on significance tests (Score test), or on information theory (Akaike Information Criterion), while the third method employed a Bayesian argument (Bayesian Model Averaging).

**Results:**

All three methods resulted in a similar parsimonious survival model. Three of the covariates previously used in the multivariate model were not included in the final model suggested by the three approaches. When comparing the parsimonious model to the previously published model, there was evidence of less variance in the main survival estimates.

**Conclusions:**

The variable selection approaches considered in this study allowed building a model based on significance tests, on an information criterion, and on averaging models using their posterior probabilities. A parsimonious model that balanced these three approaches was found to provide a better fit than the previously reported model.

## Introduction

The methods commonly used in selecting variables for multivariate models can be overshadowed with objective statistical tests while others, in contrast, grant several subjective decisions to the researcher. There are risks associated with methods that rely too heavily on either objectivity or subjectivity. Most notably for the latter is the concept of researcher bias, which is a process in which the researcher influences the results of an analysis by forcing certain variables into or out of regression models typically based upon associations found in previous studies or logic determined by causal pathways [1]. For example, statistical tools, such as stepwise regression and collinearity diagnostics, could suggest a variable be dropped from further analysis in a multivariate model, but the researcher may decide to ignore the suggestion and force the variable into the model. Researcher bias has the ability to impact the accuracy and precision of conclusions output from data analyses.

Conversely, some variable selection processes allow for very little, if any, influence on which variables are kept in or dropped from multivariate analyses. Such processes include using statistical significance tests (e.g., Wald) in a stepwise fashion [2], [3], in which variables are selected and/or deleted from an analysis using a pre-specified significance level (p-value). In essence, the researcher inputs all variables of interest (which may already have some associated researcher bias) into a statistical software program, and runs a stepwise selection process. As a result, the computer program outputs a model that only includes variables that are statistically significant at the pre-specified significance level. There are many theoretical reasons why this approach may perform poorly in selecting potential confounders [4]–[10]. In this case, some subjective influence over which variables are selected may be preferred.

While some researchers may opt to utilize only one method in their analyses, there seems to be an underutilized complementary nature between objective statistics and subjective decisions when determining which multivariate model is the best representation of the data. It has been shown that there exists an inverse relationship between having too many or too few variables in a model; while the former results in high variance or uncertainty, the latter results in more bias [11], or equivalently, inflated Type II error versus inflated Type I error. Thus, a simplistic and parsimonious model that fits the data well is preferred.

In this investigation, data were made available by both the Department of Defense (DoD) and the Department of Veterans Affairs (VA) in order to further examine a multivariate model (the “full” model) that has previously been published [12]. The goal of the present analysis was to compare time to all-cause mortality and clinical outcomes associated with HIV treatment and care between patients from the HIV Atlanta VA Cohort Study (HAVACS) and the US Military HIV Natural History Study (NHS) cohort, adjusted for possible confounders. Here we detail how using a mixture of commonly used hypothesis-testing statistics concurrently with information-theory approaches for model building can facilitate the detection of a preferred, parsimonious model. Alternatively, a Bayesian approach that combines several possible models is also described and compared to the other two approaches.

## Methods

### Ethics Statement

The NHS cohort has been approved by the Institutional Review Board (IRB) centrally (Uniformed Services University of the Health Sciences, Bethesda, MD) and at each participating center (Walter Reed National Military Medical Center, Bethesda, MD; Naval Medical Center, Portsmouth, VA; San Antonio Military Medical Center, TX; Naval Medical Center, San Diego, CA; and Tripler Army Medical Center, Honolulu, HI). Written consent was obtained from each patient. The HAVACS cohort has been approved by Emory University’s IRB and the Atlanta VA Medical Center Research and Development Committee. The HAVACS cohort does not require patients’ written consent as it has an IRB-approved waiver of consent.

### Study Participants

Data were collected from two cohorts: the HAVACS and the NHS. The NHS has enrolled over 5000 beneficiaries since 1986 into a prospective, multicenter observational study of HIV-infected active duty military personnel and other military beneficiaries living with HIV. The NHS cohort characteristics have been previously described [13]. The HAVACS includes all HIV-positive veterans seen for care at the Atlanta VA Medical Center since 1982 (n>3900). The cohort characteristics of HAVACS have been previously described [14]. Data are prospectively collected for both cohorts and are used for clinical care and research purposes.

Patients in both cohorts were eligible for inclusion in this analysis if they had an HIV diagnosis and began highly active antiretroviral therapy (HAART) treatment between January 1, 1996 and June 30, 2010. A total of 1199 NHS patients and 1065 HAVACS patients were followed from their recorded HAART initiation (HI) date through all-cause mortality or the date of last data entry. Separate internal analyses have determined that only 43 of the total >3900 HAVACS patients were in the NHS cohort, so the effect of any overlap between the patients examined in this analysis is negligible.

### Variable Definitions

A HAART regimen was defined as the use of three or more antiretroviral medications, one of which has to be a protease inhibitor (PI), a non-nucleoside reverse transcriptase inhibitor (NNRTI), an integrase inhibitor, or an entry inhibitor. A participant’s HI date was the date of the first HAART regimen that lasted greater than one month, utilizing an intention to treat format. Information regarding the participant’s age at HI, sex, race, year of HIV diagnosis, time from HIV diagnosis to HI, viral load and CD4 count at HI (within three months prior to HAART start date), history of chronic hepatitis B and C co-infection, and previous AIDS-defining illness (ADI) excluding CD4<200, and previous ARV use (mono or dual NRTI) were also analyzed. There were no time-updated variables utilized in these analyses.

### Statistical Analysis for Full Model

Potential covariates were selected based on previous literature regarding influences on survival of patients living with HIV in the presence of HAART [15]. Causal diagrams guided by literature were also drawn to ensure all selected variables would, in theory, reduce bias rather than induce bias in the analysis [10], [16]. The covariates were then screened using bivariate analyses and dropped from further inclusion in multivariate models if their crude association’s p-value with the outcomes were greater than 0.5. Using an epidemiological modeling approach [16], [17], all the remaining variables and their interactions with the study’s exposure were combined in a multivariate model (the “global” model) and assessed for collinearity. Using the concept of hierarchically well-formulated models [16], the interaction terms were first assessed for elimination from the model using a likelihood ratio test for significance. The non-interaction, confounding terms were then assessed utilizing the change-in-estimate approach [4]–[7], [9], [10]. This approach is similar to a backwards selection procedure, and a variable was kept in the multivariate model if its exclusion resulted in an “important” change to the main exposure variable’s coefficient estimate. A 10% change cut-off was deemed “important” in this analysis. Covariates that remained after these procedures were utilized throughout all analyses.

Kaplan-Meier curves and Cox proportional hazards models were fitted to investigate time from HI to all-cause mortality by cohort. Specific to survival analysis, the proportional hazards (PH) assumptions were gauged for each covariate utilizing a consensus of three different approaches: graphical, goodness of fit, and extended Cox modeling [17], [18]. All covariates of interest satisfied the PH assumption. Thus, we can assume that the model including all remaining covariates (the previously published “full” model), or any subset of covariates, fit the data [19].

### Statistical Analysis for Parsimonious Model

The Score test [20] is an hypothesis-testing statistic that is similar to commonly used likelihood ratio tests and Wald tests. All three are equivalent in large samples [21]. The Score statistic is less computational (requiring only evaluations under the null), and can be used as the basis for eliminating or including variables in such models. It is important to note that only nested models can be compared under this approach.

The Akaike Information Criterion (AIC) provides a practical and versatile way to identify a parsimonious model from a set of competing final models, by adding a penalty term proportional to the number of parameters in the model. This penalty term guards against overfitting. The AIC procedure yields an objective mathematical tool to determine parsimony in model construction and is free of subjective judgments [22]. Based on a penalized likelihood, the AIC allows comparison of non-nested models as well. Its benefits have been extensively described, particularly in the context of ecological and genetic study designs [23]–[28]; however, AIC procedures are applicable to other research topics, such as survival of patients living with HIV [29]. The AIC value was manipulated into two other related statistics; Akaike weights (*w_i_*) and evidence ratios (ER) [30]. An Akaike weight is found by taking the proportion of the exponentiated AIC differences between model *i* and the model with the smallest AIC value to the sum of all these differences in all *K* subset models:
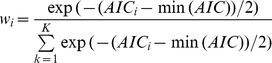
(1)


The resulting value indicates the probability that the chosen *i* model is the best to represent that data among the whole set of *K* candidate models (those that were used when calculating the denominator in equation 1 above).

Evidence ratios are a small extension to Akaike weights – it directly compares the weights of two models *i* and *j*:
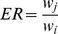
(2)


The yielded value depicts the likelihood that model *j* is better or worse than model *i* to best represent the data given the two models selected.

An important remark regarding the previous approaches is that they are two-step procedures: first select a model (variable selection), and second perform statistical tests given (conditional on) the selected model being correct. This approach underestimates the variability in the data, by ignoring the uncertainty in the selected models. Moreover, in practice, two models can fit the data equally well but provide different estimated effect sizes [31]. Bayesian model averaging addresses these issues in a Bayesian context. Several models are considered, and the posterior distribution of the quantity of interest is the average of the posterior distributions under each model considered, weighted by their posterior model probabilities [32]. More specifically, given the data *D*, the posterior distribution of the parameter of interest *β* is given by
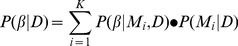
(3)


Where *M_1_,…M_K_* are the models considered, and *P*(*M_i_|D*) is the posterior probability of the model *M_i_* given the data.

In a survival data context, several Cox models *M_1_,…M_K_* are fitted, and *β* is the hazard ratio of interest [33]. The best *K* models in terms of the Bayesian information criterion (BIC) are selected using an efficient leaps and bound algorithm [34], and the posterior hazard ratio is estimated. One can also estimate a conditional posterior hazard ratio (HR), conditional on that particular covariate be included in all *K* models considered. The conditional HRs for each variable are obtained after averaging the posterior HRs only for the models containing that particular variable, and therefore are more likely to have nonzero HRs, while the unconditional HRs are obtained by averaging over all top *K* selected models. The result is that unconditional HRs are expected to be closer to zero than conditional HRs. We emphasize the conditional approach to Bayesian model averaging because it strikes a balance between an unconditional approach and stepwise selection – it averages over the posterior models that include the particular variable, allowing for the other variables to be included or not in the model.

Analyses were conducted with SAS 9.2 (SAS, Cary, NC, USA) and R (R Development Core Team (2008). R: A language and environment for statistical computing. R Foundation for Statistical Computing, Vienna, Austria). SAS and R code can be found as Figure S1.

## Results

### Hypothesis-Testing Approach

Twelve covariates were initially selected from the databases to be included in the modeling process. These potential confounders were selected due to their influences on the survival of patients living with HIV in the presence of ARV therapy [35]. After bivariate analyses, one variable, year of HIV diagnosis, was dropped. The global model was constructed to include the 11 remaining covariates and their interactions with the main exposure variable, which denotes the cohort of each patient. After collinearity assessments and backwards elimination processes, all interaction terms were dropped from the list of potential covariates. The change-in-estimate procedure detected subset models; however, none of these models performed better than a model including all remaining covariates with regards to the precision of the exposure’s effect. Thus, the full model included only 12 covariates, one of which was the main exposure.

### Information-Theory Approach

**Figure 1:**
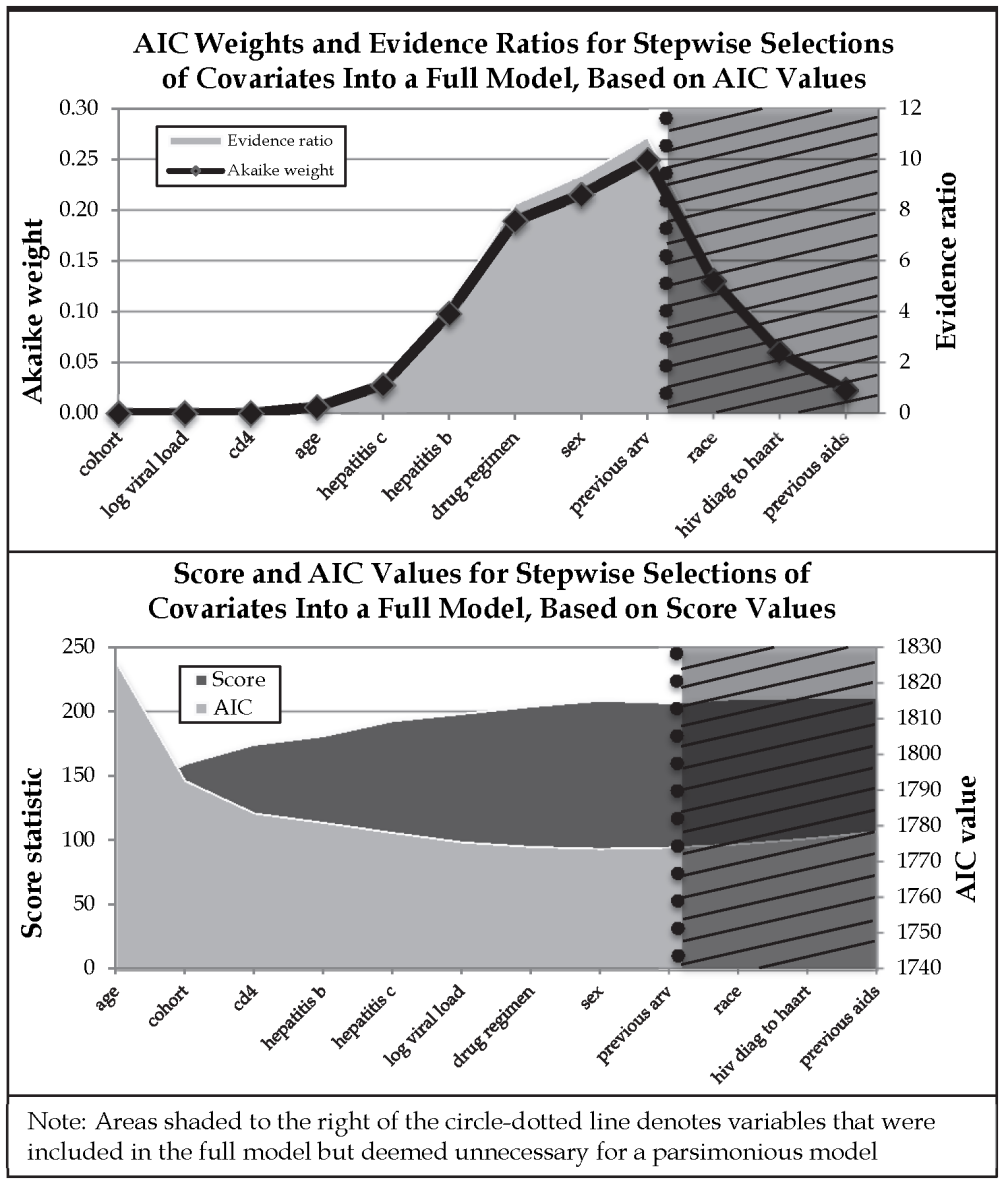
Comparing Subset Models to a Full Model in Order to Detect a Potential Parsimonious Model. Top panel title: AIC Weights and Evidence Ratios for Stepwise Selections of Covariates Into a Full Model, Based on AIC Values. Bottom panel title: Score and AIC Values for Stepwise Selections of Covariates Into a Full Model, Based on Score Values. Note: Areas shaded to the right of the circle-dotted line denotes variables that were included in the full model but deemed unnecessary for a parsimonious model.

Instead of halting the variable selection process, Score tests and AIC comparisons were then implemented on the full model to determine if a better fitting and more parsimonious model existed. All 12 variables were cumulatively added to a multivariate model in two fashions: by the largest incremental change in AIC value and by the largest incremental change in Score statistic. Both of these statistics detected the existence of a parsimonious model; in fact, the same parsimonious model was found using both methods to add covariates to the model (Figure 1).

Akaike weights and ERs were computed for each subset model and found that a parsimonious model that did not include race, time from HIV diagnosis to HI, and previous ADI was 10.9 times more likely than the full model to be the best model to represent the data, given the chosen set of subset models. The Score tests similarly suggested that this parsimonious model was the best model.

The full model controlling for all 12 covariates concluded that patients in NHS were 57% less likely to die during follow-up compared to patients in HAVACS (HR 0.43, 95% CI 0.27, 0.70; p = 0.0005). However, the parsimonious model that dropped race, previous ADI, and time from HIV diagnosis to HI, concluded that NHS patients were 58% less likely to die during follow-up compared to patients in HAVACS (HR 0.42, 95% CI 0.27, 0.65; p = 0.0001). The minimal change in the main effect estimate between the models can be attributed to the lack of information the three variables (race, previous ADI, and time from HIV diagnosis to HI) brought to the full model. In fact, after exploring the distributions of these three variables, there was evidence for a lack of racial diversity in the HAVACS cohort (78.8% were African-American/Black), a lack of previous ADI events in the NHS cohort (3.4%), and there were similar time periods between HIV diagnosis and HI in the entire sample (median: 4.7 months in the NHS; 5.0 months in the HAVACS, p = 0.6774).

### Bayesian Model Averaging Approach

**Figure 2:**
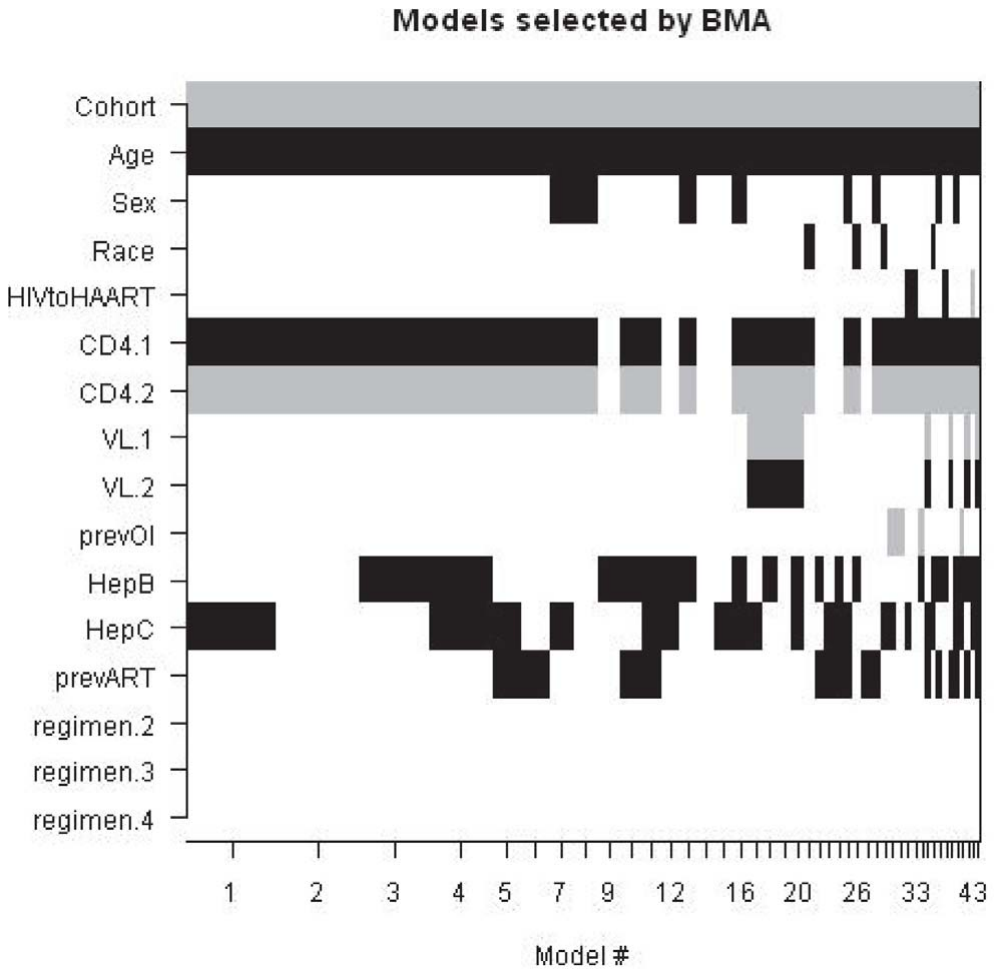
Models Considered by Bayesian Model Averaging, in Order of Posterior Probability. Variables Positively (Negatively) Associated With Outcome are in Grey (Black).

The model with the highest approximate posterior probability was a model including cohort, age, CD4, and hepatitis C. This model represented only 11.3% of the total posterior probability, while the top five models had a cumulative posterior probability of 42.2%, indicating a moderately high model uncertainty. Figure 2 depicts the top 44 models (columns) in terms of their posterior probability that were used for model averaging (*K* = 44). Variables (rows) positively correlated with the outcome appear in black, while those negatively correlated with the outcome appear in grey. Column width for each column (model) is proportional to the posterior probability of that model.

Table 1 also presents the posterior hazard ratios and the conditional hazard ratios, conditional on the corresponding variable being included in the model. The posterior probability is the probability that the corresponding variable is correlated with the outcome. There is close agreement between the AIC-based variable selection approach and the Bayesian model averaging. The variables race, time from HIV diagnosis to HI, and previous ADI were dropped from the parsimonious model and similarly had low posterior probabilities (4.3%, 3.2%, and 3.4%, respectively). The only discrepancy is the variable initial HAART regimen, which was not dropped in the AIC approach, but has a zero posterior probability under the Bayesian approach. It is of note that within the AIC approach, the difference in AIC values for a model including initial HAART regimen and one without the variable was merely 0.46 (1773.57 vs. 1774.04, respectively). Regarding the selected variables, a general remark is that the confidence intervals in the parsimonious model are usually tighter, due to decreased uncertainty introduced by the excluded variables. The effects of hepatitis B and C and previous ARV use appear more impactful in the AIC-based approach relative to the Bayesian approach (HR 1.86, 1.63, and 1.33 versus HR 1.33, 1.24, and 1.08, respectively). Interestingly, the two approaches give very similar results conditional on the variables being included in the model. Thus, the biostatistical tools used in these analyses detected problematic variables and removed them to form a better fitting model that collinearity tests alone could not identify. Otherwise, the conclusions that the full model suggests are similar to those of the parsimonious model, thus confirming the AIC’s detection of a model that results in minimal information loss.

**Table 1:** Comparison of All-Cause Mortality Cox Regression Models Between NHS and HAVACS Patients Based on Three Approaches: Hypothesis-Testing, Information-Theory, And Bayesian Model Averaging, 1996 – 2010, n = 1,727.

	Hypothesis-testing		Information-theory		Bayesian model averaging				
	"Full" model		"Parsimonious" model			Posterior		Conditional posterior	
Covariate	HR	95% CI	HR	95% CI	Selected (%)	HR	95% CI	HR	95% CI
Cohort, NHS vs. HAVACS	**0.43**	**0.27, 0.70**	**0.42**	**0.27, 0.65**	**100.0%**	**0.39**	**0.25, 0.61**	**0.38**	**0.25, 0.61**
**Demographics**									
Age at HAART initiation	**1.06**	**1.04, 1.08**	**1.06**	**1.04, 1.08**	**100.0%**	**1.06**	**1.04, 1.08**	**1.06**	**1.04, 1.08**
Sex, female vs. male	2.64	0.64, 10.80	2.58	0.63, 10.55	14.1%	1.14	0.50, 2.64	2.60	0.64, 10.59
Race, AA vs. other	1.20	0.80, 1.79	–		4.3%	1.00	0.91, 1.11	1.16	0.79, 1.74
**Medical history (prior to HAART initiation)**									
HIV diagnosis to HAART initiation	1.00	0.99, 1.01	–		3.2%	1.00	1.00, 1.00	1.00	0.99, 1.01
Viral load at HAART initiation, log copies/mL					9.8%				
*<2.60*	0.46	0.19, 1.15	0.47	0.19, 1.15		0.93	0.55, 1.57	0.46	0.08, 2.87
*2.60–3.99*	1.46	0.91, 2.33	1.46	0.91, 2.33		1.04	1.01, 1.36	1.48	0.27, 8.25
*≥4.00*	ref		ref			ref		ref	
CD4 at HAART initiation, cells/mL					85.0%				
*<200*	1.28	0.79, 2.07	1.30	0.82, 2.06		1.36	0.84, 2.20	1.43	0.45, 4.60
*200–349*	0.60	0.34, 1.03	0.61	0.35, 1.05		0.69	0.38, 1.24	0.64	0.19, 2.26
*≥350*	ref		ref			ref		ref	
Previous AIDS-defining illness^a^	0.95	0.62, 1.43	–		3.4%	1.00	0.92, 1.08	0.92	0.61, 1.39
Chronic hepatitis B co-infection	**1.89**	**1.10, 3.25**	**1.86**	**1.09, 3.20**	44.5%	1.33	0.65, 2.73	1.90	1.10, 3.26
Hepatitis C co-infection	**1.60**	**1.08, 2.38**	**1.63**	**1.12, 2.39**	49.0%	1.24	0.75, 2.06	1.55	1.06, 3.26
Previous antiretroviral use	1.40	0.95, 2.07	1.33	0.93, 1.92	23.6%	1.08	0.78, 1.49	1.38	0.96, 1.98
**Initial HAART regimen**					0.0%				
Non-nucleoside reverse transcriptase inhibitor	ref		ref						
Boosted protease inhibitor	**1.78**	**1.10, 2.86**	**1.74**	**1.08, 2.81**					
Unboosted protease inhibitor	1.28	0.86, 1.90	1.29	0.87, 1.91					
Other	0.72	0.25, 2.01	0.71	0.25, 1.99					

Abbreviations: AA - African -American/Black ; AIDS – acquired immune deficiency syndrome; CD4 – cluster of differentiation 4; CI – confidence interval; HAART - Highly Active Antiretroviral Therapy; HAVACS - HIV Atlanta VA Cohort Study; HR – hazard ratio; NHS - US Military HIV Natural History Study; ref – referent group.

Notes: Bold hazard ratios and 95% confidence intervals are significant at the *P*<0.05 level.

a 1993 definition, exclusive of CD4<200.

## Discussion

The AIC has been reported to be a better tool in the field of model selection than traditional hypothesis-testing [22], [28]. In certain instances, stepwise variable selection based on hypothesis-testing may lead to different final models, depending on the order the variables are considered (e.g., forward, backward). The information-theoretic approach utilized by the AIC statistic often yields consistent results and does not depend on the order of which variables are selected [19], [28], [36]. In this example, an information-theoretic approach was balanced with a limited use of hypothesis-testing, which resulted in a parsimonious model that was nearly 11 times more likely than the full model to be the best choice. The utilization of AIC values or Score tests resulted in the same parsimonious model, which suggests that any additional exploration for a better-fitting model to only traditional hypothesis-testing may result in less variance in its estimates. The variables dropped from the full model to create the parsimonious model were chosen to be included in the initial multivariate analyses due to their cited influences on the survival of patients living with HIV while on HAART; nevertheless, due to the unique characteristics of the cohorts used in this analysis, they did not necessarily need to be controlled for in a model. Furthermore, the excluded variables were already being controlled for indirectly; in particular, previous AIDS diagnosis was partly captured by the CD4 count at HAART initiation variable (i.e., AIDS is usually associated with lower CD4).

Stepwise methods, such as the AIC approach used in this analysis, are two-stage procedures. First, a model that shows evidence of fit is selected, and then, conditional on this model being correct, one estimates the hazard ratios associated with the variables selected, and significance tests are performed. The Bayesian approach provides an alternative paradigm in which several models are considered at the same time, and the posterior parameters are simply weighted averages of the estimates from each model, with weights given by the posterior probability of each model given the data. One benefit of this approach is that it provides a coherent framework for distinguishing between lack of power to detect a significant effect, and a lack of correlation between the covariate and the outcome [33].

These analyses emphasize that while no single model is perfect, some are more useful than others. Similarly, these analyses suggest that only using the common hypothesis-testing methods could result in a model including variables that introduce excessive variance in the estimates. The goal is to have an expansive and rich “toolbox” of methods than can guide a researcher to a more useful model. When multiple approaches lead to the same or similar results, the conclusion is considerably strengthened and ultimately variance is diminished. The statistical tools used in this analysis are also applicable to other regression-type models. Further research should be employed on alternative datasets and data types to explore situations in which this multiple-approach method does not result in the same or similar final models as well as models aiming to make accurate predictions of an outcome.

## Conclusions

Given the advent of methodological developments and the availability of statistical software, exploring several options for variable selection may prove beneficial when building complex multivariate models. Our analyses demonstrated such an amalgamation that resulted in a better-fitting, parsimonious multivariate model than what would have been reported by utilizing only one approach. We propose the usage of the same or similar robust, multi-approach methodology in an attempt to detect a preferred parsimonious model in other analyses.

## Supporting Information

Figure S1 SAS and R code.
(PDF)
