# Supplementary material for: Combining Epidemiologic and Biostatistical Tools to Enhance Variable Selection in HIV Cohort Analyses
Source: PLoS One. 2014 Jan 29;9(1):e87352. doi: 10.1371/journal.pone.0087352 (PMC3906149; doi:10.1371/journal.pone.0087352)
Supplement: Figure S1 — SAS and R code. (PDF) [file pone.0087352.s001.pdf]

Figure S1. SAS and R code

I. Full to parsimonious model in SAS

```
**dsn is the dataset that includes all clean covariates used in analyses;

proc phreg data = dsn;
	class cohort (ref=last) cd4hart_3cat (ref=last) logvloadhart_3cat (ref=last)
	regimen4 (ref="NNRTI") ;
	model surv_death*died(0) = cohort ageathart sex aa_race dgtohart cd4hart_3cat
	logvloadhart_3cat prevoi hepbhart hepchart prev_art regimen4  /rl ;
title1 "Full model after collinearity";
run;

proc phreg data = dsn;
	class cohort (ref=last) cd4hart_3cat (ref=last) logvloadhart_3cat (ref=last)
	regimen4 (ref="NNRTI") ;
	model surv_death*died(0) = cohort ageathart sex cd4hart_3cat logvloadhart_3cat
	hepbhart hepchart prev_art regimen4  /rl ;
title1 "Parsimonious model";
run;
```

**Score, Wald, Likelihood Ratio, and Wald statistics are included in PROC PHREG output. Variables were dropped in a manual fashion by re-running the above code to include or exclude specific variables as defined in the associated manuscript.

II. AIC-based Variable Selection in R

```
##  bb is a dataframe with components (“surv_death”, ”died”, "cohort”, ”ageathart”,
## ”sex”, ”aa_race”, ”dgtohart”, ”cd4hart_3cat”, ”logvloadhart_3cat”, ”prevoi”,
## ”hepbhart”, ”hepchart”, “prev_art”, ”regimen4”)

## fit the full model
library(survival)
cox_full = coxph(Surv(surv_death,died) ~ cohort + ageathart + sex + aa_race +
dgtohart +  cd4hart_3cat + logvloadhart_3cat + prevoi + hepbhart + hepchart +
    prev_art + regimen4, data = bb)
summary(cox_full)

## stepwise selection based on AIC
library(MASS)
cox_AIC  = stepAIC(cox_full,direction="both")
summary(cox_AIC)

## check the proportional hazards assumption
cox.zph(cox_AIC)
```

III. Bayesian Model Averaging (BMA) in R

```
## fit the BMA model
library(BMA)
cox_bma = bic.surv(bb[,c("cohort","ageathart","sex","aa_race","dgtohart",
"cd4hart_3cat","logvloadhart_3cat","prevoi","hepbhart","hepchart",
   "prev_art","regimen4")],bb$surv_death,bb$died,
factor.type=TRUE,strict=FALSE)
summary(cox_bma).

## conditional posterior means and standard deviations, conditional on including the
## corresponding variable in the model
cox_bma$condpostmean
cox_bma$condpostsd

## plot the models selected
imageplot.bma(cox_bma,col=c("black","grey","white"))
```
